# Downregulation of mPGES-1 Expression *via* EGR1 Plays an Important Role in Inhibition of Caffeine on PGE_2_ Synthesis of HBx(+) Hepatocytes

**DOI:** 10.1155/2015/372750

**Published:** 2015-10-11

**Authors:** Yan Ma, Xiaoqian Wang, Nanhong Tang

**Affiliations:** ^1^Fujian Institute of Hepatobiliary Surgery, Fujian Medical University Union Hospital, 29 Xinquan Road, Fuzhou 350001, China; ^2^Key Laboratory of Ministry of Education for Gastrointestinal Cancer, Research Center for Molecular Medicine, Fujian Medical University, Fuzhou 350001, China

## Abstract

We investigated the mechanism of caffeine in influencing HBx(+) hepatocytes to synthesize PGE_2_. The inhibitory effect of caffeine on hepatocyte proliferation increased with increasing caffeine concentrations (200–800 *μ*M) and treatment times (1–7 days), which was first observed at the second test time point (caffeine treatment for 4 days). The inhibition of caffeine on the growth of HL7702-HBx and HepG2-HBx cells was most obvious at 800 *μ*M caffeine and at caffeine treatment for 7 days. The PGE_2_ secretion and the expression of mPGES-1 and EGR1 were downregulated, whereas PPAR*γ* expression was upregulated. The mPGES-1 promoter activity of HBx(+) hepatocytes decreased more significantly than that of HBx(−) hepatocytes. Moreover, the expression of EGR1 and PPAR*γ* changed more significantly in HBx(+) hepatocytes cultured for 12 to 24 hours in the presence of 5 mM caffeine. This limited success may be attributed to caffeine releasing the binding of HBx and PPAR*γ* and furthermore affecting the mPGES-1 expression by EGR1 in HBx(+) hepatocytes. The results indicate that caffeine could effectively reduce PGE_2_ synthesis in HBx(+) hepatocytes by specifically blocking the PPAR*γ*-EGR1-mPGES-1 pathway, thereby providing a new evidence of molecular biology for the hypothesis that drinking coffee is beneficial to HBV-infected patients.

## 1. Introduction

Hepatocellular carcinoma (HCC) is the third leading cause of death worldwide. An estimated 748300 new liver cancer cases and 695900 cancer deaths have been recorded worldwide in 2008 [1]. In China, most HCC patients are infected by hepatitis B virus (HBV), which undergoes a process from hepatitis to liver cirrhosis to HCC. This process is one of the significant differences between HCC and other malignant tumors. HBV, a noncytopathic specific double-stranded DNA virus, could cause acute and chronic hepatitis [2]. Hepatitis B virus x protein (HBx) has various biological functions that could be simultaneously expressed in the nucleus and cytoplasm of hepatocytes. HBx could promote the occurrence and progress of liver cirrhosis and HCC [3].

Prostaglandin E2 (PGE_2_) is one of the important products with the most biological activity synthesized by cyclooxygenase. PGE_2_ is significantly increased in malignant tumor tissues and plays a critical role in HBV virus infection, as well as in the occurrence and progress of HCC [4, 5]. The synthesis of PGE_2_ is higher in hepatocytes with positive HBx [6]. PGE_2_ could increase the expression of oncogene in the mRNA and protein levels. The EP_4_/GS/AC/CREB/NF-*κ*B molecular signaling pathways promote the growth and invasion of cancer cell [7]. Reduction of PGE_2_ could effectively inhibit the invasion of HCC [4].

Relevant epidemiologic studies have shown that the incidence of HCC for the people who drink coffee every day is 30%–80% lower than those who do not. Caffeine has the same protective effect for chronic hepatitis B virus carriers and high-risk populations developing liver cancer [8]. A survey on 63,000 Chinese Singaporeans between the ages of 45 and 74 years conducted by the National University of Singapore shows that consumption of caffeine is negatively related to the incidence of HCC. The incidence of HCC for people who drink three cups of coffee or above every day is significantly reduced by 44% [9]. A comparison between the 109 HBV carriers and 125 subjects in the control group collected by the Prince of Wales Hospital of Hong Kong shows that drinking coffee in moderation could reduce nearly half of the risk of HCC [8]. Some studies have shown that caffeine could effectively inhibit PGE_2_; however, the specific mechanism remains unclear [10]. Moreover, whether caffeine has a particular role in the regulation of PGE_2_ synthesis in hepatocytes with HBx expression for the study on HCC associated with HBV infection is the focus of our further investigation. Exploring the effect of caffeine on PGE_2_ synthesis pathways may provide a theoretical basis for the study on preventive strategies using caffeine in HBV-infected patients.

## 2. Materials and Methods

### 2.1. Material

The recombinant plasmid pcDNA3.0-HBx was constructed by our laboratory [11]. HL7702 cells (a human hepatic cell line, Institute of Biochemistry and Cell Biology, Shanghai, China), previously established HL7702-HBx cells (stable HBx expression by transfection with pcDNA3.0-HBx) [12], HepG2 cells (a human hepatocarcinomal cell line, HB-8065, ATCC, VA), and HepG2-HBx cells (stable HBx expression by transfection with pcDNA3.0-HBx) were grown in DMEM containing 10% fetal bovine serum at 37°C in a humidified atmosphere containing 5% CO_2_. The microsomal prostaglandin E synthase-1 (mPGES-1) promoter luciferase reporter plasmid, pGL3B-628 (−628 to +1), and cyclooxygenase-2 (COX-2) promoter luciferase reporter plasmid, pGL3B-COX-2, were constructed by our laboratory [13]. Caffeine (Sigma, St. Louis, MO) was dissolved in culture medium (4 × 10^3^ mM) and a certain volume was drawn to be added to the cell well for reaching the required final concentration.

### 2.2. Proliferation Assay

Briefly, 5 × 10^3^ of HL7702, HL7702-HBx, HepG2, and HepG2-HBx cells were, respectively, seeded in different wells of 96-well plates and incubated overnight. Caffeine solution was, respectively, added to the different wells with a final concentration (200, 400, or 800 *μ*M); the same amount of medium without caffeine was added to the control wells. 24 hours later, the wells of the first test point (caffeine treatment for 1 day) were washed two times by PBS and incubated with 20 *μ*L 3-(4,5-dimethylthiazol-2-yl)-2,5-diphenyltetrazolium bromide (MTT, 5 mg/mL, Sigma, St. Louis, MO) for 4 h. Formazan crystals were subsequently dissolved in 150 *μ*L dimethyl sulfoxide (DMSO). The absorbance of the solution was measured at 490 nm and detected using the Bio-Tek lQuant Universal Microplate Spectrophotometer (Bio-Tek Instruments, Inc., Winooski, VT). For the second (caffeine treatment for 4 days) and third (caffeine treatment for 7 days) test points, the cell wells were, respectively, replaced with new culture medium (containing different concentrations of caffeine) at the third day and the sixth day after cultivation. Each treatment was repeated in triplicate.

### 2.3. PGE_2_ Analysis

Twenty-four hours after 5 × 10^3^ cells/well were plated onto 96-well plates, the caffeine solution was, respectively, added with a final concentration (800 *μ*M); the same amount of medium without caffeine was added to the control wells. One, 4, and 7 days later, the supernatant was, respectively, collected and centrifuged briefly. The amount of PGE_2_ in the supernatant was determined using EIA Kit (Cayman Chemical, Ann Arbor, MI) according to the manufacturer's protocol. Parallel cells were harvested and counted. All assays were performed three times.

### 2.4. Dual-Luciferase Reporter Assay

2 × 10^5^ of HL7702, HL7702-HBx, HepG2, and HepG2-HBx cells were, respectively, seeded in different wells of 24-well plates and incubated overnight. The supernatant was removed and replaced with serum-free medium, and DNA transfection solution containing promoter luciferase reporter plasmid and Lipofectamine 2000 (Life Technologies, Carlsbad, CA) was added to the cells, in accordance with the manufacturer's recommendations. Eight hours later, the caffeine solution was, respectively, added with a final concentration (800 *μ*M) and it continued to be cultured for 48 h. The supernatant was removed and the cell lysates were detected for intracellular luciferase activity using Dual-Luciferase Reporter Assay System (Promega Corporation, Madison, WI) on a luminometer (Orion II Microplate Luminometer, Berthold Detection Systems, Germany) following the manufacturer's recommendations. The relative luciferase units (RLU) were obtained by comparison with control, which was set to 1. Each transfection was performed in triplicate and the data were expressed as the mean ± SD of three separate experiments.

### 2.5. Western Blotting

1 × 10^5^ of HL7702, HL7702-HBx, HepG2, and HepG2-HBx cells were, respectively, seeded in different wells of 6-well plates and incubated overnight. Caffeine solution was added with different final concentrations and then the plates were incubated for different time according to the experimental requirement. Cell protein was extracted by conventional method. Each treatment was repeated in triplicate. A total of 40 *μ*g of protein was subjected to 12% sodium dodecyl sulfate-polyacrylamide gel electrophoresis (SDS-PAGE) and electrophoretic transfer to a polyvinylidene fluoride (PVDF) membrane (Millipore, Billerica, MA). Protein blots were incubated separately with a panel of specific antibodies from Santa Cruz Biotechnology, Inc. (Santa Cruz, CA), which included anti-mPGES-1 (1 : 1000, sc-12269), anti-COX-2 (1 : 1000, sc-19999), anti-early growth response 1 (EGR1) (1 : 1000, sc-110), anti-peroxisome proliferator-activated receptor gamma (PPAR*γ*) (1 : 1000, sc-7273), and anti-*β*-actin (1 : 4000, sc-47778) overnight at 4°C and then incubated with different horseradish peroxidase- (HRP-) conjugated secondary antibody at room temperature for 1 h. Visualization of the immunoreactive proteins was performed by chemiluminescence kit (BeyoECL Plus, Beyotime, Shanghai, China). Intensities of band signals were quantified using the densitometric software Quantity One (Bio-Rad, Hercules, CA) and the relative intensity to internal control (*β*-actin) was calculated. All measurements were repeated in triplicate.

### 2.6. Statistical Analysis

The data were repeated at least three independent times and expressed with mean ± SD unless otherwise indicated. Assays for characterizing phenotype of cells and expression difference were analyzed by one-way analysis of variance (ANOVA) using SPSS 13.0 software package (SPSS, Inc., Chicago, IL). *P* < 0.05 denoted a statistically significant difference.

## 3. Results

### 3.1. Effect of Caffeine on Hepatocyte Proliferation and Secretion of PGE_2_

**Figure 1:**
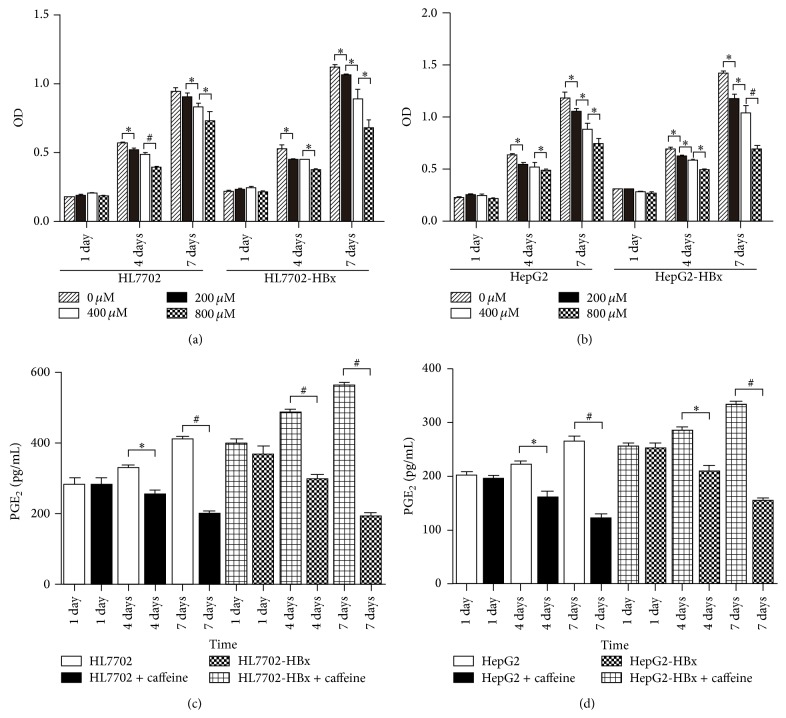
Effect of caffeine on hepatocyte proliferation and synthesis of PGE_2_. ((a) and (b)) Cell proliferation at different caffeine concentrations (0, 200, 400, and 800 *μ*M) and different caffeine treatment days (1, 4, and 7 days). The group data represent the mean ± SD (*n* = 3); ∗ denotes a statistically significant difference (*P* < 0.05); # denotes a statistically significant difference (*P* < 0.001). ((c) and (d)) PGE_2_ level in the supernatant of different cells in caffeine treatment (800 *μ*M) for 7 days. The group data represent the mean ± SD (*n* = 3); ∗ denotes a statistically significant difference (*P* < 0.05); # denotes a statistically significant difference (*P* < 0.001).

Caffeine with different concentrations was used to treat four strains of hepatocyte (HL7702, HL7702-HBx, HepG2, and HepG2-HBx). Figures 1(a) and 1(b) show that the inhibitory effect of caffeine on hepatocyte proliferation increased with increasing caffeine concentrations (200–800 *μ*M) and treatment times (1–7 days), indicating that caffeine inhibits cell proliferation in a dose- and time-dependent manner. The inhibition activity of caffeine on the four strains of hepatocyte was first to be observed at the second test time point (caffeine treatment for 4 days) (*P* < 0.05). Meanwhile, the inhibition of caffeine on HL7702-HBx and HepG2-HBx cells was most obvious at the concentration of 800 *μ*M and at the third test time point (caffeine treatment for 7 days), which was significantly higher than that on HL7702 and HepG2 cells (*P* < 0.05), respectively. Therefore, we chose 800 *μ*M as the optimum concentration of caffeine for subsequent experiments.

Four strains of cells were cultured in a medium containing caffeine (800 *μ*M) for 7 days. The cell supernatant was collected to test the secretion of PGE_2_. Figures 1(c) and 1(d) show that the amounts of PGE_2_ secreted from the four strains of cells were gradually reduced after culture with caffeine (800 *μ*M) for 1, 4, and 7 days (*P* < 0.05). Compared with the first test time point (caffeine treatment for 1 day), the amounts of PGE_2_ secreted from the four strains of cells cultured at the third test time point (caffeine treatment for 7 days) were reduced by 29.4% (HL7702), 47.5% (HL7702-HBx), 38.6% (HepG2), and 43.0% (HepG2-HBx), respectively. This result indicates that the inhibition of caffeine on PGE_2_ secreted from HL7702-HBx and HepG2-HBx cells was higher than that secreted from the HL7702 and HepG2 cells.

### 3.2. Effect of Caffeine on the Expression of COX-2 and mPGES-1 in HBx Positive Hepatocyte

**Figure 2:**
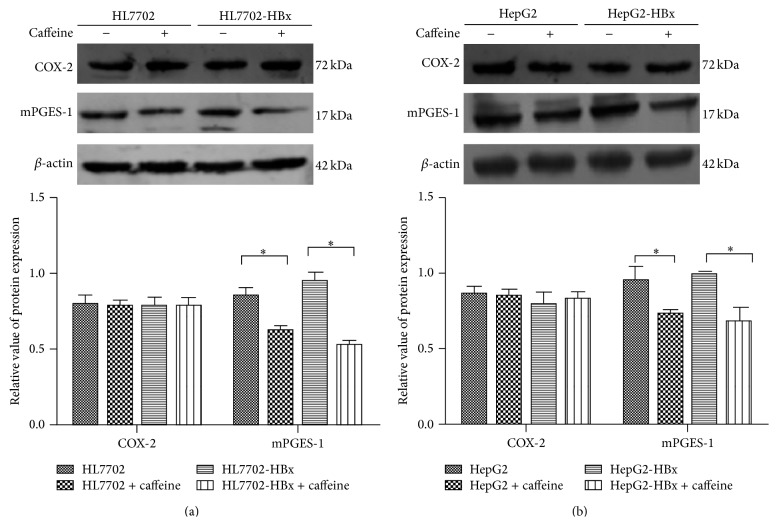
Effect of caffeine on the expression of COX-2 and mPGES-1 in HBx positive hepatocyte. (a) Representative immunoblots for COX-2, mPGES-1, and *β*-actin in HL7702 and HL7702-HBx cells. (b) Representative immunoblots for COX-2, mPGES-1, and *β*-actin in HepG2 and HepG2-HBx cells. The group data represents the mean ± SD (*n* = 3). The densitometry data were normalized to *β*-actin. Note: ^∗^ *P* < 0.05, statistically significant difference compared to HL7702 cells (a) or HepG2 cells (b).

The synthesis of PGE_2_ covers the effects of a series of enzymes among which COX-2 and mPGES-1 are the rate-limiting enzymes that play key roles [14]. In the presence of 800 *μ*M of caffeine for 7 days, the cell proteins of HL7702, HL7702-HBx, HepG2, and HepG2-HBx cells were, respectively, extracted for detecting the expressions of COX-2 and mPGES-1.  Figure 2 shows that caffeine had no insignificant effect on COX-2 expression of the four strains (*P* > 0.05). The mPGES-1 protein expression with the addition of caffeine was significantly lower than that without caffeine (*P* < 0.05). The mPGES-1 protein expression of HL7702-HBx and HepG2-HBx cells with the addition of caffeine was significantly lower than that of HL7702 and HepG2 cells (*P* < 0.05).

### 3.3. Effect of Caffeine on the Transcriptional Activity of COX-2 and mPGES-1 Gene Promoter in HBx Positive Hepatocytes

**Figure 3:**
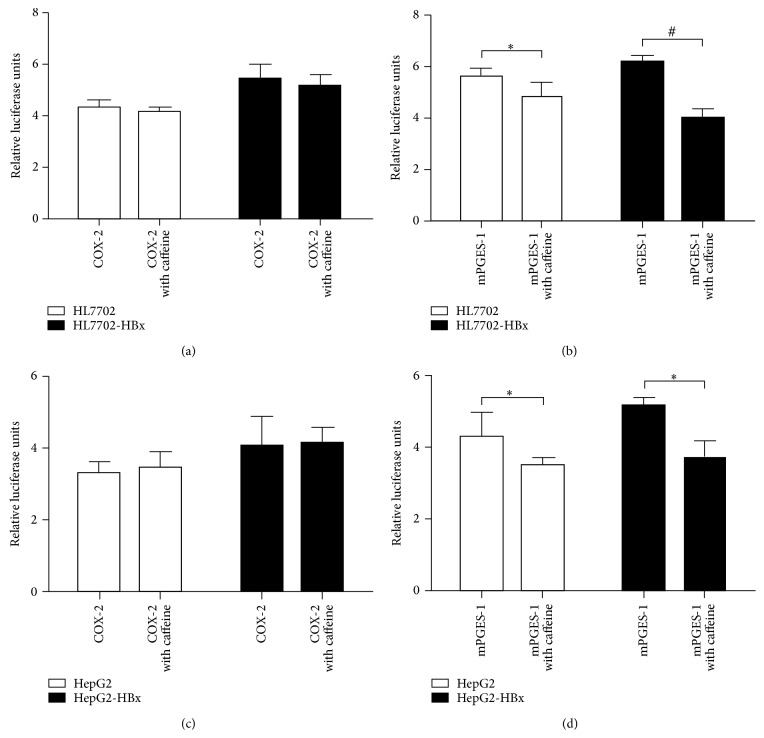
Effect of caffeine on the transcriptional activity of COX-2 and mPGES-1 gene promoter in HBx positive hepatocytes. (a) The COX-2 promoter activity (RLU value) in HL7702 and HL7702-HBx cells. (b) The mPGES-1 promoter activity (RLU value) in HL7702 and HL7702-HBx cells. (c) The COX-2 promoter activity (RLU value) in HepG2 and HepG2-HBx cells. (d) The mPGES-1 promoter activity (RLU value) in HepG2 and HepG2-HBx cells. Cells were cotransfected with 1 *μ*g of pGL3B-COX-2 (COX-2 promoter luciferase reporter plasmid) or pGL3B-628 (mPGES-1 promoter luciferase reporter plasmid) and 100 ng pRL-TK, and pGL3-basic served as the negative control. ^∗^ *P* < 0.05 or ^#^ *P* < 0.001 versus HL7702 or HepG2 cells (RLU was set to 1, *n* = 3).

We further investigated whether caffeine caused the change in protein through transcriptional link. Approximately 800 *μ*M of caffeine was used to treat HL7702, HL7702-HBx, HepG2, and HepG2-HBx cells to detect the fluorescent activity of the gene after the mPGES-1 and COX-2 promoter had been transiently transfected for 48 hours.  Figure 3 shows that addition of caffeine had no effect on the transcriptional activity of the COX-2 promoter of four strains (*P* > 0.05), but the transcriptional activity of the mPGES-1 promoter was downregulated more significantly with the addition of caffeine than that without caffeine (*P* < 0.05). After the addition of caffeine, the activity of the mPGES-1 promoter in the HL7702-HBx and HepG2-HBx cells decreased more significantly than that in HL7702 and HepG2 cells (*P* < 0.05). This result indicates that caffeine could inhibit the activity of the mPGES-1 promoter, thereby affecting the expression of mPGES-1.

### 3.4. Inhibition of Caffeine on the Expression of mPGES-1 through EGR1 in HBx Positive Hepatocytes

**Figure 4:**
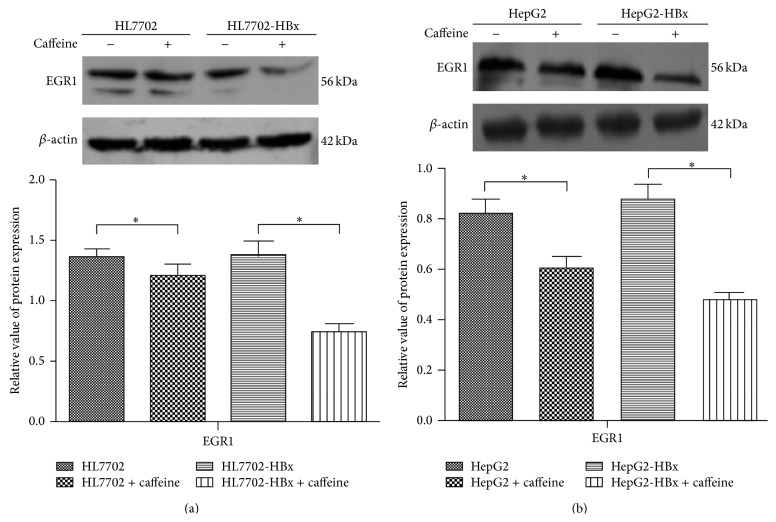
Inhibition of caffeine on the expression of mPGES-1 through EGR1 in HBx positive hepatocytes. (a) Representative immunoblots from three independent studies for EGR1 in HL7702 and HL7702-HBx cells treated with 800 *μ*M caffeine or not for 7 days. The densitometry data were normalized to *β*-actin. ^∗^ *P* < 0.05 (*n* = 3), statistically significant difference compared to HL7702 cells. (b) Representative immunoblots from three independent studies for EGR1 in HepG2 and HepG2-HBx cells treated with 800 *μ*M caffeine or not for 7 days. The densitometry data were normalized to *β*-actin. ^∗^ *P* < 0.05 (*n* = 3), statistically significant difference compared to HepG2 cells.

Our previous research showed that HBx could upregulate the transcription of mPGES-1 promoter through EGR1, thereby enhancing the expression of mPGES-1 in promoting the hepatocytes to secrete PGE_2_ [13]. The role of EGR1 is very important in this process. Therefore, caffeine may inhibit the expression of mPGES-1 through the EGR1 pathway; that is, caffeine could inhibit the synthesis pathway of HBx-EGR1-mPGES-1-PGE_2_. To verify this tentative idea, we detected the EGR1 expression of HL7702, HL7702-HBx, HepG2, and HepG2-HBx cells in the presence of caffeine (800 *μ*M).  Figure 4 shows that the EGR1 protein expression of the four strains of cells was downregulated (*P* < 0.05), indicating that caffeine could downregulate mPGES-1 expression by inhibiting the EGR1 expression in the hepatocytes. Meanwhile, we observed that HL7702-HBx and HepG2-HBx cells in response to caffeine stimulation were more significant than HL7702 and HepG2 cells (*P* < 0.05).

### 3.5. Effect of Caffeine on EGR1 Expression through PPAR*γ* in HBx Positive Hepatocytes

**Figure 5:**
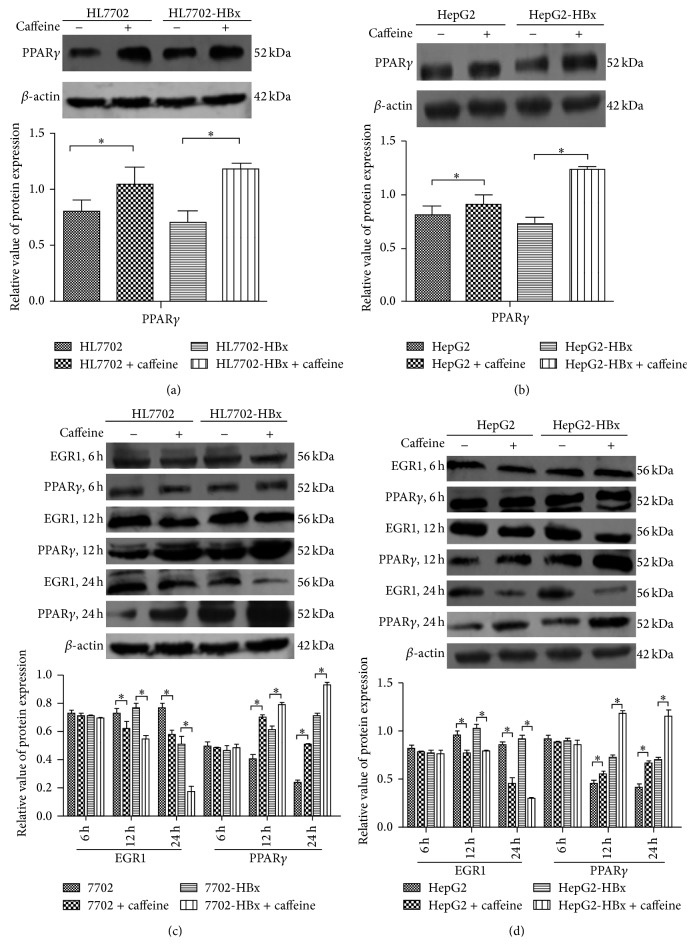
Effect of caffeine on EGR1 expression through PPAR*γ* in HBx positive hepatocytes. (a) Representative immunoblots from three independent studies for PPAR*γ* in HL7702 and HL7702-HBx cells treated with or without 800 *μ*M caffeine for 7 days. The densitometry data were normalized to *β*-actin. ^∗^ *P* < 0.05 (*n* = 3), statistically significant difference compared with HL7702 cells. (b) Representative immunoblots from three independent studies for PPAR*γ* in HepG2 and HepG2-HBx cells treated with or without 800 *μ*M caffeine for 7 days. The densitometry data were normalized to *β*-actin. ^∗^ *P* < 0.05 (*n* = 3), statistically significant difference compared with HepG2 cells. (c) Representative immunoblots from three independent studies for EGR1 and PPAR*γ* in HL7702 and HL7702-HBx cells treated with or without 5 mM caffeine for 6, 12, and 24 h. The densitometry data were normalized to *β*-actin. ^∗^ *P* < 0.05 (*n* = 3), statistically significant difference compared to HL7702 cells. (d) Representative immunoblots from three independent studies for EGR1 and PPAR*γ* in HepG2 and HepG2-HBx cells treated with or without 5 mM caffeine for 6, 12, and 24 h. The densitometry data were normalized to *β*-actin. ^∗^ *P* < 0.05 (*n* = 3), statistically significant difference compared to HepG2 cells.

Previous studies showed the interaction of proteins between HBx and PPAR*γ*, which inhibited the nuclear orientation of PPAR*γ* and the DNA binding function and affects the expression of the relative growth-inhibited gene regulated by PPAR*γ* [15]. To verify whether caffeine could affect EGR1 expression by PPAR*γ* to block the secretion of PGE2 from hepatocytes caused by HBx, we used 800 *μ*M caffeine (a lower dose) to treat HL7702, HL7702-HBx, HepG2, and HepG2-HBx cells for 7 days. The results showed that the expression of PPAR*γ* was increased, and the expression of PPAR*γ* increased more significantly in the presence of HBx (*P* < 0.05) (Figures 5(a) and 5(b)). Approximately 5 mM caffeine (a higher dose) was further used to treat these cells for 6, 12, and 24 hours. Figures 5(c) and 5(d) show that the expression of EGR1 and PPAR*γ* did not significantly change after 6 hours of cultivation. After 12 and 24 hours, the expression of PPAR*γ* in the case with addition of caffeine was significantly higher than that without caffeine. The increase was more significant over time (*P* < 0.05). Meanwhile, the EGR1 expression with the addition of caffeine was significantly lower than that without caffeine; the decrease was more significant over time (*P* < 0.05). The changing trend of EGR1 and PPAR*γ* expression was more significant in the presence of HBx.

## 4. Discussion

PGE_2_ is one of the important products with the most biological activities synthesized by cyclooxygenase. Studies have shown that PGE_2_ is significantly increased in malignant tumor tissue and could promote the growth of tumor cells [16]. Therefore, inhibition of PGE_2_ has become one of the valuable research directions against inflammation to cancer. Currently, many drugs or compounds that could inhibit cells in producing PGE_2_ have been found. These drugs could destroy or affect various enzymes expression in the generation process of PGE_2_ [17–19]. The biosynthesis of PGE_2_ is regulated by phospholipase A2, cyclooxygenase (COX), and mPGES-1. Inhibition of the above-mentioned enzymes could prevent the synthesis of PGE_2_. Previous studies have shown that the expression level of COX-2 in tumor cells increased, and specific COX-2 inhibitor could inhibit tumor cell proliferation, induce apoptosis, and enhance the sensitivity of tumor cell to chemotherapy drugs. However, improper use of COX-2 inhibitor could cause kidney damage and increase the incidence of cardiovascular disease and thrombus [20].

In our previous study, we found that HBx protein could regulate the transcriptional activity of mPGES-1 promoter through EGR1. During this process, PPAR*γ* plays an important role in inhibiting the combination of EGR1 and mPGES-1 promoter to prevent the transcription of mPGES-1 [13]. An interaction exists between HBx and PPAR*γ*, which could affect the expression of the relative growth-inhibited gene regulated by PPAR*γ* by inhibiting the nuclear orientation of PPAR*γ* and DNA binding function to release the inhibition on cell growth [15]. 15d-PGJ_2_, a ligand of PPAR*γ*, was used to interfere in this process. The result showed that the combined action of 15d-PGJ_2_, PPAR*γ*, and EGR1 could regulate the secretion of PGE_2_ in the cell through mPGES-1. For hepatocytes expressed by HBx, the presence of 15d-PGJ_2_ breaks the relationship between HBx and PPAR*γ*. Activated PPAR*γ* could inhibit the combination of EGR1 and mPGES-1 promoter to prevent the transcriptional activity of mPGES-1, thereby inhibiting the occurrence of PGE_2_. This finding indicates that compound intervention in the relationship between PPAR*γ* and EGR1 for affecting HBx in the expression of mPGES-1 is an effective way. Interestingly, although the relationship between proinflammatory mediator PGE_2_ and liver disease has been given significant attention, studies on the influence of HBV infection on PGE_2_ synthesis and the relevant intervention are rare. Therefore, we use two hepatic cell lines with different backgrounds, as well as their derived cell lines with HBx expression, to observe the characteristics of caffeine interfering in PGE_2_ synthesis in HBx(+) hepatocytes.

Coffee is a common drink. Various biological activities of caffeine, the main constituent of coffee, have been widely studied. In the prevention and treatment of diseases, caffeine has a positive effect. Some studies have shown drinking coffee could reduce the risk of liver cancer. A study has shown that caffeine could enhance the sensitivity of hepatocytes to 15d-PGJ_2_, PGE_2_ specific inhibitor, by upregulating the expression of PPAR*γ* receptor in hepatocytes. Caffeine could also promote the degradation of SMAD2 and inhibit phosphorylation of SMAD1 and SMAD2 [21]. The above-mentioned effects of caffeine downregulate the hepatic fibrosis-related connective tissue growth factor of inflammatory cytokines induced by TGF-*β* (CCTG), thereby inhibiting the progress of hepatic fibrosis. The interaction between caffeine and mPGES-1 has not been reported yet. In this paper, we found that the mPGES-1 and EGR1 expression and mPGES-1 promoter activity in the hepatocytes treated by caffeine are significantly lower than those of the group without caffeine. The expression of PPAR*γ* was significantly higher than that of the group without caffeine. These changes are more significant in HBx(+) hepatocytes. Therefore, we speculated that caffeine has an effect similar to 15d-PGJ_2_, which could release the binding of HBx and PPAR*γ* in DBA hinge regions and activate PPAR*γ*, to play the role of PPAR*γ* in the inhibition of cell growth. Moreover, we also found that caffeine has insignificant effect on COX-2 expression and promoter transcription of PGE_2_ biosynthetic enzymes. That is, caffeine does not have side effects similar to COX-2 inhibitor-like drugs in inhibiting HCC growth. In addition, the potential adverse side effects caused by inhibition of normal levels of PGE_2_ produced by hepatocytes must be taken into consideration. Based on our current experiments* in vitro* using low concentration of caffeine, we speculated that the side effects of PGE_2_ inhibition by caffeine may be fewer; however, the exact effects should be verified in animal experiments in the future.

Apparently, our study reveals that caffeine could effectively reduce PGE_2_ synthesis in HBx(+) hepatocytes by specifically blocking the PPAR*γ*-EGR1-mPGES-1 pathway and delay the effect of PGE_2_ in promoting HCC growth, which provides a new evidence of molecular biology for the hypothesis that drinking coffee is beneficial to HBV-infected patients.
